# Food Insecurity and Hunger in Rich Countries—It Is Time for Action against Inequality

**DOI:** 10.3390/ijerph16101804

**Published:** 2019-05-21

**Authors:** Christina M Pollard, Sue Booth

**Affiliations:** 1 Faculty of Health Science, School of Public Health, Curtin University, Perth 6845, Australia; 2 College of Medicine & Public Health, Flinders University, Adelaide 5000, Australia; sue.booth@flinders.edu.au

**Keywords:** food insecurity, hunger, developed countries, Sustainable Development Goals, social determinants, inequality, food banks

## Abstract

Household food insecurity is a serious public health concern in rich countries with developed economies closely associated with inequality. The prevalence of household food insecurity is relatively high in some developed countries, ranging from 8 to 20% of the population. Human rights approaches have the potential to address the structural causes, not just the symptoms of food insecurity. Despite most developed countries ratifying the Covenant on Economic, Social and Cultural Rights over 40 years ago, food insecurity rates suggest current social protections are inadequate. The contemporary framing of the solution to food insecurity in developed countries is that of diverting food waste to the hungry to meet the United Nations Sustainable Development Goals agenda (Goals 2 and 12.3). An estimated 60 million people or 7.2% of the population in high income countries used food banks in 2013. Although providing food assistance to those who are hungry is an important strategy, the current focus distracts attention away from the ineffectiveness of government policies in addressing the social determinants of food insecurity. Much of the action needed to improve household food security falls to actors outside the health sector. There is evidence of promising actions to address the social determinants of food insecurity in some developed countries. Learning from these, there is a strong case for government leadership, for action within and across government, and effective engagement with other sectors to deliver a coordinated, collaborative, and cooperative response to finding pathways out of food insecurity.

## 1. Introduction

Household food insecurity is a serious public health concern in rich countries with developed economies [1]. For example, in Australia, Canada, Europe, New Zealand, the United Kingdom, and the United States of America (US), improving household food and nutrition security is a public health priority. Food insecurity is costly, has wide-reaching consequences, and its effects extend beyond vulnerable populations. The two main ways of addressing food security in developed countries continue to be measures to respond to poverty including welfare entitlements and food relief [2]. As governments retreat from the issue, third sector organizations step in to deliver services to people in need, evidenced by the rapid proliferation of food banks and charitable food services. As expected, food assistance does little to address the underlying causes of food poverty and insecurity [3]. Clearly the response in developed countries is not working. There are tangible solutions to the problem, what is missing in many countries is the political will to fully acknowledge the problem and take the effective action.

Human rights-focused approaches have the potential to address the impact of government action or inaction, including the structural causes, not just the symptoms, of social inequities. The right to food is bound under international law in Article 25.1, “*Everyone has the right to a standard of living adequate for the health and well-being of himself and of his family, including food, clothing, housing and medical care and necessary social services, and the right to security in the event of unemployment, sickness, disability, widowhood, old age or other lack of livelihood in circumstances beyond his control.*” p. 76 [4]. Enshrined in the International Covenant on Economic, Social and Cultural Rights (ICESCR) Article 11 [5] consenting nation states are obligated to respect, protect and fulfil their commitments, see General Commitment number 12 [5]. Adopted in 1966 [6] with entry into force in 1976, many developed countries have ratified ICESCR including: Australia (1975); Canada, United Kingdom, Great Britain, Northern Island (1976); Japan (1979); and Belgium (1983). The US is a notable exception, signing in 1977 but not yet ratified, and continues to express resistance towards economic and social rights [7]. 

Countries who are ratified are subject to investigations on their current situation by the Special Rapporteur on the Right to Food. Progress on the realisation of the right to food can be very slow, even in rich countries. Nearly 40 year after ratification, the 2012 Canadian review by the Special Rapporteur, found 57% of people living on social assistance were food insecure and concluded that Canadian cash transfers were insufficient for an adequate standard of living [8]. Although Canada’s promotion of labour market participation as a strategy to overcome poverty was commended, it was recommended that their minimum wage legislation should be a ‘living wage’ [8]. Housing costs were noted as a key reason people were compelled to use food banks. 

A sense of urgency to address food insecurity is implicit in the Global Sustainable Development Goals (SDG) which seek actions to realise human rights by 2030, and are determined to end poverty and hunger, in all their forms and dimensions….. Goal 2 (2.1) has a target “*to end hunger and ensure access by all people, in particular the poor and people in vulnerable situations, including infants, to safe, nutritious and sufficient food all year round.*” [9].

## 2. How You Define Food Insecurity Shapes the Response

How you define and measure a problem influences how you respond to it. Before effective action to address food insecurity can be taken, there is a need to agree on a definition of food security and understand its determinants. A clear definition provides the context for action and assists with identifying the desired outcomes. Since the early 1990s there have been numerous definitions of food insecurity with meanings and actions differing when applied at global, domestic, household, or individual levels. This paper refers to food and nutrition security at the household and individual level in rich countries.

The Committee on World Food Security in 2012, recognising that the response to food insecurity involved multidisciplinary actors who need to speak the same language, sought a standard definition. Food and nutrition security, existing *“when all people at all times have physical, social and economic access to food, which is safe and consumed in sufficient quantity and quality to meet their dietary needs and food preferences, and is supported by an environment of adequate sanitation, health services and care, allowing for a healthy and active life”* p. 8 [10] was adopted. Definitions change and reflect what is socially acceptable at the time. The 2012 definition encompassed some broader aspects of food insecurity, such as sanitation, health services, and care, but moved away from tenets which emphasise non-emergency provision [11], avoiding unorthodox procurement practices (scavenging, stealing, or other coping strategies), social justice, democratic decision making, community self-reliance [12], or providing diets rather than just food [11]. The determinants as well as the extent of the problem are important. Food insecurity is the therefore the limited or uncertain availability of nutritionally adequate or safe foods or limited or uncertain ability to acquire foods in socially acceptable ways [13].

The determinants of food and nutrition insecurity as well as the extent of the problem are important considerations when defining action. Between and within country differences are important considerations, including, but not limited to: geographical differences (e.g., urban versus rural); chronicity and severity levels; political, economic and social drivers; historical government positions; climate impact; and how these factors have changed over time.

## 3. The Framing of the Issue Determines the Response

The way a problem is framed, or publically portrayed, also shapes the way society responds. Framing food security issues in ways that resonate with the beliefs, priorities and needs of different audiences can mobilize support for action. Within country social inequalities, particularly poverty, lead to food insecurity [2], therefore it would make sense that the problem framing should be in terms of how to address social and economic inequity. Two main frames dominate the way developed countries define the problem of food insecurity and the way government and other key stakeholders’ respond—societal benefit and food waste mitigation.

Societal benefit frames household food security in terms of how both individuals and society benefit when all members of society are food secure. Countries with high levels of food security benefit socially, economically, environmentally, and politically. The economic cost of food insecurity is not routinely reported in developed nations however estimates to date suggest they are substantial. Costs related to food insecurity in 2011 in the US were ~A$167.5 billion related to lost productivity, public education expenses, avoidable healthcare costs, and the cost of charity to keep families fed [14]. Food insecurity is associated with a range of physical and mental health issues which contribute significantly to healthcare costs, for example cardiovascular disease [15] and obesity [16]. There is a clear relationship between housing instability, food insecurity and access to health care amongst low income families [17]. Reducing food insecurity would see improvements in health, employment, productivity, and economic viability, and reductions in health care costs. This framing should inform arguments about the need for an urgent response to food insecurity in developed countries, as the cost of inaction is likely to be far more deleterious [14]. The complexity of social disadvantage contributing to poverty, through exposure to adversity throughout the life course and often across generations, should inform the responses to food insecurity [18,19].

Food waste mitigation, “*Waste not want not. Toward zero hunger. Food bank as a green solution to hunger*”, is the contemporary framing used by the 2019 Global Foodbanking Network which frames foodbanks as the solution to hunger (SDG 2) and the environmental impact of food waste (SDG 12.3) [20]. Governments, the commercial sector, the voluntary sector, and social entrepreneurs are increasingly framing food waste diversion to the hungry as a social, economic, and environmental win:win:win [21]. There are significant economic, environmental, and social impacts of food surplus and waste, and countries need to ensure sustainable food systems to remain food secure [21]. The strongest solution to the problem is prevention that is, reducing food surplus at its source through holistic changes in the food system. The framing of the issue of food surplus and waste is currently focussed on recovery as the primary solution that is, reusing waste food for human consumption, an insufficient remedy for long term food insecurity and for food waste [21]. For example, in Australia and the UK, the voluntary sector partners with food businesses to divert food [2,22,23], and in France, food waste redistribution to charities is legislated [24]. Although these approaches may provide some food for relief agencies, unfortunately, the conflating of the two issues does not solve the fundamental and complex problems of either of them [23]. The problem of food surplus/waste distribution is the nature of the food distributed and the manner in which it is provided.

Framing food and nutrition security action within broad policy discourses (for example achieving the SDGs or economic and social policy reform) can generate commitment to act. The United Nation’s World Health Organization and Food and Agriculture Organization Driving commitment for nutrition within the UN Decade of Action on Nutrition policy brief frames the argument that taking action on improving nutrition is a win-win option for many sectors and works to achieve at least 12 of the 17 SDGs. Designing frames to resonate with the people who can influence action is important [25].

Framing can be further enhanced to resonate with the people who influence action. For example, financial policy makers would likely be interested in societal benefits in terms of economic rationale (e.g., cost to health systems), civil society groups would be interested in ‘the human right to adequate food and to freedom from hunger’, and the vulnerability of children to malnutrition may resound with all audiences [25].

Reframing and focusing food insecurity to address the broader sustainable development issues of supporting human rights and sustainable development to create an equitable and prosperous society, will have much broader impacts than the current focus on redistributing food waste. For example, societal benefits in terms of economic rationale (e.g., cost to health systems, workforce productivity) would likely be of interest to financial policy makers; ‘the human right to adequate food and to freedom from hunger’ may inspire civil society groups; and the focus on vulnerability of children to malnutrition may resound with all audiences [25].

## 4. The Scale of the Problem of Food Insecurity

A double burden of malnutrition (high rates of undernutrition (including wasting, stunting, and micronutrient deficiencies) co-existing alongside overweight, obesity, and diet-related non-communicable diseases) is commonplace in many countries. In 2017, the absolute number of people affected by undernourishment or chronic food deprivation was estimated to be 821 million and 9 billion adults were overweight or obese. At the same time, 151 million children aged under 5 years were stunted and 38 million were either overweight or obese. Undernutrition contributes to 3.1 million (45% of total) deaths in children under five every year. The UN Decade of Action on Nutrition 2016–2025 (http://www.un.org/nutrition) aims to trigger intensified action to end hunger and eradicate all forms of malnutrition worldwide. Undernourishment, severe food insecurity, and malnutrition is more prevalent in developing economies, 90% of the worlds stunted children live in 36 countries with the highest level of chronic undernutrition. Taking action in these countries is clearly the highest priority to achieve the SDG [26].

## 5. How Big Is the Problem of Food Insecurity in Developed Countries?

“*No Data, No Problem, No Action.*”, the title of a paper by Friel et al. (2009), captures the crux of the matter in terms of defining the problem of food insecurity in developed countries [27]. Relatively hidden in most developed countries, the population prevalence of food insecurity is largely unreported due to a lack of routine measurement and use of non-comparable measures. Food insecurity is closely associated with poverty and as some countries have no official government statistics, household food insecurity estimations are made using proxy measures such as national poverty lines (50 to 60% of median income) [28]. Estimated food insecurity prevalence is unexpectedly high in some rich countries, for example: Australia and Japan (21.7% of households, ~4.6 million people and 15.7%, ~19.8 million, respectively in 2012—based on 50–60% of the national poverty line); Canada (7.7%, ~1.9 million in 2007/8); the European Union (8.7% or 43.6 million when 27 countries are included); and the US (15% of the population, ~50 million) [28]. 

Canada and the US regularly monitor household food insecurity, while in other countries, such as the UK, it has been the rapid rise of food banks that has drawn attention to the issue [1]. Food insecurity monitoring using comparable measures should be a mandatory requirement across all countries as without the compulsory requirement national comparable estimates are at risk. Canada, who has monitored food insecurity since 2005, had some jurisdictions opt out of voluntary monitoring, undermining their ability to produce national estimates [29]. The Food and Agriculture Organization (FAO) of the United Nations supports the use of comparable measures of food insecurity to capture its magnitude, severity and causes [30]. The FAO supports an enabling environment for a rapid response to hunger through better data to: shape policies and programs; increase political commitment; support effective co-ordination and evidenced-based decision making. There is an urgent need for most developed countries to commit to using comparable measures and for most, significant effort is needed to meet this recommendation. This surveillance of within- and across-country food insecurity is crucial intelligence for the government and other sector’s decision making regarding actions to address food insecurity, and a fundamental requirement for reporting against the SDGs.

## 6. The Responses to Food Insecurity

Most developed countries respond to food insecurity through the provision of food assistance delivered by the voluntary sector, with very limited government support [31]. Addressing the social determinants of food insecurity is the exception, for example, Norway’s political agenda focuses on agricultural support, food pricing regulation, and universal social security [2]. Food assistance is usually in the form of food banks, pantries, parcels, and soup kitchens delivered by the voluntary or charitable sector. The US has embedded government funded food assistance programs: the Supplemental Nutrition Assistance Program (SNAP) of the Food Stamp Act of 1964, provided ~14% of Americans support for household food purchases at a cost of an estimated A$70 billion annually in 2018 [32]; the Special Supplemental Nutrition Program for Women, Infants and Children (WIC) serves 7 million participants a month at a cost of A$5.95 billion in 2016 [33]. 

Low cost food has been made available through food banks in the US since the late 1960s [34], which began opening in Europe 20 years later, and are now present in all Organisation for Economic Co-operation and Development (OECD) member states [31]. About 60 million people or 7.2% of high income country populations used them in 2013 [28]. The Global Foodbanking Network comprises over 800 food banks in 31 countries [19]. The proportion of the population accessing food banks is relatively high in some developed countries, for example, 12% of the US population (37 million people) in 2009 and 6% (19 million) of those living in the EU used foodbanks in 2011 [28]. It appears that food banks are now a permanent fixture in the response to food insecurity in developed countries, but at what cost?

Countries with relatively low public social spending have greater numbers of foodbank users. For example, the US has 12% of the population using foodbanks and spends 19.7% of Gross Domestic Product (GDP) on social expenditure whereas Belgium has ~1.9% total population using food banks and spent 29.6% of GDP on social expenditure in 2011 [28]. The rapid growth in food banks and public appeals for food donations or money for food suggest a normalisation of food aid in the UK [35] and other developed countries [36]. Despite food banks, food charity, and government programs, food insecurity is a growing problem in rich countries, so what is going wrong?

Each developed country has its own social protection systems or social welfare safety net which, due to reconstructions and cut backs to basic entitlements has meant that food banks have “*become secondary extensions of weakened social safety net*” p. 648 [37]. The inadequacy of developed countries’ social protection systems is rendering people vulnerable to food insecurity, as demonstrated by increased food insecurity rates in these countries. In fact, “*Social protections systems, not the least unemployment and child benefits must be recalibrated to take into account the real cost of living and ensure adequate food for all, without compromising on other essentials*” p. X, the Special Rapporteur on the Right to Food [38]. Clearly, food banks can provide emergency food assistance but do not, in and of themselves, offer pathways out of food insecurity in developed nations [31]. 

The experience of being food insecure and seeking food assistance in rich countries can have negative impacts on the individual as it is traumatic, stressful, and detrimental to one’s health and wellbeing [39,40]. In all societies, “*to be mentally healthy you must value and respect yourself”* p. 65 [41]. People who use food assistance in rich countries say they experience stigma, shame, and hopelessness [35,42,43,44,45]. Shame is a powerful emotion related to feeling foolish, stupid, ridiculous, inadequate, defective, incompetent, awkward, exposed, vulnerable, and insecure, based on seeing oneself negatively in the eyes of the other [46]. It is the inequality within rich countries that fosters feelings of inferiority, even before needing to seek food assistance. Independence is a core value in Western culture, people who need help to meet a basic need, such as food, are viewed as dependent and dependency is humiliating [47]. This is understandable as needing food assistance and the ways it is currently delivered is not considered socially acceptable, nor should it be in a wealthy country [48]. Another concern is that food banks and pantries strongly influence user’s diets, yet are unable to support an adequate dietary intake [49,50]. Trying to address household food insecurity with community-based food interventions is not effective when solutions likely lie upstream in social protection policies [1].

## 7. What Should or Could Be Done and by Whom 

A decade ago, the notion that ‘equality is better for everyone’ was eloquently expressed by Wilkinson and Pickett (2009) who asserted that to improve the quality of life in rich countries the focus must shift from building material standards and economic growth to finding ways to improve the psychological and social wellbeing of whole societies, essentially by reducing within country inequality [41]. 

**Figure 1:**
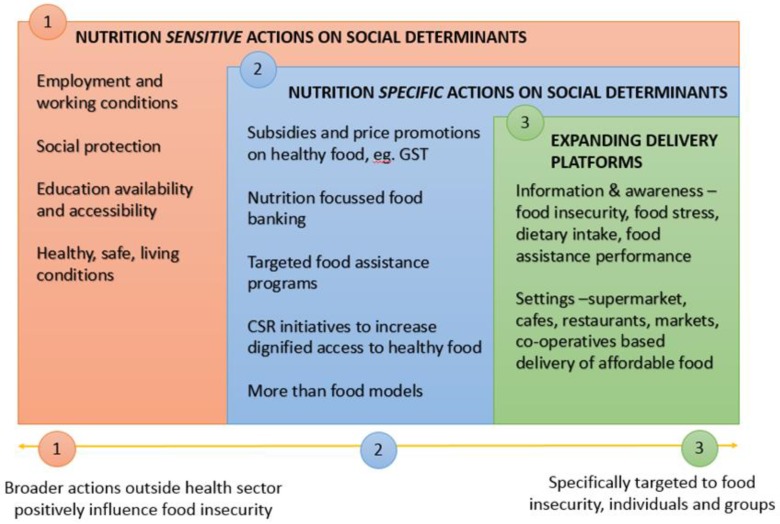
Typology of multi-sectoral actions on food and nutrition security (Adapted from Figure 8 p. 45 Discussion Paper Addressing the Social Determinants of Non-communicable Diseases [52]).

When looking for what could or should be done to address food and nutrition security in developed countries, the discussion paper on addressing the social determinants of non-communicable diseases (NCD) provides a useful framework for multi-sectoral action outside the health sector [51]. Influenced by Friel et al.’s (2015) suggestions to address inequities in healthy eating [52], we adapted the framework for NCD action to one addressing food and nutrition security in developed countries, see Figure 1. Importantly, we have re-ordered the action focus based on potential to reduce food insecurity.

There is evidence to suggest some key actions to take to achieve the SDGs related to address food insecurity in developed countries. The prerequisites for action on the social determinants of food and nutrition security are high-level political commitment, governance mechanisms to facilitate and coordinate multi-sectoral responses, and robust structures for monitoring, evaluation, and accountability. As much of the required action needed to improve food and nutrition security is to be taken by actors outside the health sector, strong advocacy is needed to create cross-sector, cross-government engagement to build a shared understanding of the problem of food insecurity, outline potential actions, and delegations of responsibility. The initial advocacy focus to support the argument for cross-sector action for societal benefit, would include continuing to measure the problem and its impact and using this information to engage various sectors. There is likely to be benefit in high level government leadership bringing together key stakeholders for human development benefit, second to commercial interests. Fine-grained measurement, multilevel monitoring systems, action on the social and environmental determinants of health, and inclusive systems of governance are required to address food insecurity [27].

Political commitment to address nutrition can be created and strengthened over time through strategic action. Baker et al.’s (2018) review of factors that generated political commitment for nutrition action identified the following important drivers, irrespective of country: effective nutrition actor networks; strong leadership; civil society mobilisation; supportive political administrations; societal change and focusing events; cohesive and resonant framing; and robust data systems and available evidence [53]. Private sector interference was found to frequently undermine commitment in high-income countries.

Key actions to build food and nutrition security are in order of potential influence starting with food and nutrition sensitive, followed by food and nutrition specific actions, and lastly expanding delivery platforms. Some examples of promising actions are include:

(1) *Food and nutrition sensitive actions* include core business of non-health actors to address social determinants, including regulating employment and labour conditions, increasing access to education, challenging harmful gender norms, promoting a rights-enhancing legal environment, setting urban development policy or developing social protection programmes. Macro level changes to laws, policies and social structures can redistribute power and resources. All of the actions listed above address the determinants of food insecurity. As a matter of urgency, across government and across sector action is needed to reduce social and economic inequality. Valuable lessons can be learnt from some Scandinavian countries where the social protections systems promote equality [2] and studies from Canada and the UK focussing on reducing financial hardship [54,55]. Government policy to support access to affordable housing without compromising basic needs such as food is recommended [56].
(2) *Food and nutrition specific actions* change conditions of daily life to be those that provide food and nutrition security via interventions including laws, policies, and programmes whose primary purpose is action on the social determinants. Promising actions include: subsidies and price promotions on healthy food to ensure its affordability, for example, the Australian exemption of healthy basic foods from the Goods and Services Tax [57] and in-store price promotions; procurement policies promoting nutrition focussed food banking [58,59]; building support for the nutrition focus in targeted food assistance programs (e.g., SNAP and WIC); Corporate Social Responsibility initiatives to increase dignified access to healthy food and affordable food for all, for example, Lidl’s ‘Too Good to Waste’ boxes selling 5 kilograms of slightly damaged but edible fruit and vegetables for just £1.50 [60]; ‘More than food’ models of food assistance that provide emergency relief with integrated support services to help people find pathways out of food insecurity [61,62]. Three key principles for the food service aspect of these models are: (1) a client-centred focus; (2) empowering individuals by fostering autonomy and enabling food choice in socially acceptable ways; and (3) providing opportunities for active involvement, social connection, and broader support [63].
(3) *Expanding delivery platforms* use settings to extend the reach of the health sector and extend the reach and impact of health-related information. This includes a focus on non-charitable food-service settings for example, supermarkets, cafes, restaurants, farmers markets, co-operatives, and social enterprise models to ensure affordable food is available to people at high risk of food insecurity in non-stigmatising ways. Government monitoring and surveillance systems, independent of the food industry and the charitable food sector should be developed to contribute country level information to inform appropriate actions. At a minimum these should include using standardised and robust measures of: household food and nutrition security that captures severity and prevalence and includes children (e.g., the United States Department of Agriculture (USDA)’s 18 item U.S. Household Food Security Survey Module where appropriate or part of the suite [64]); food related measures of financial stress (e.g., food stress [65]); routine measure of dietary intake, measured height and weight, and socioeconomic status; and food assistance services performance indicators. Collectively these build information and intelligence systems to inform the delivery of targeted food insecurity interventions.

The impact of food insecurity is ultimately felt by the individual, the health system and all of society. Although not directly responsible for service delivery of the actions described above, the health sector is well placed to work with other sectors to support them to ensure effective responses to food insecurity. The three main priority actions of the health sector in developed countries to address food and nutrition insecurity are: (1) to provide the technical expertise (nutrition science, public health, food safety, and health promotion) to assist the development of food and nutrition policies to ensure interventions are nutritionally adequate and do not exacerbate health issues; (2) To contribute to the systematic monitoring and surveillance of the performance and outcomes of the comprehensive range of actions described above in terms of food security and other health outcomes; (3) Advocate on behalf of those who are rendered food insecure due to hardship and disadvantage for effective responses to food insecurity across government at all levels and stages of country development across the globe. 

There is a key role for academia to provide the evidence base and independent voice to inform and evaluate policy and programs and to challenge existing paradigms and assumptions. The *International Journal of Environmental Research and Public Health* Special Issue on Addressing Food Insecurity in Developed Countries is a good example of how the research community can come together to provide evidence to guide policy and practice [66,67,68,69,70,71,72,73,74,75,76,77,78,79,80,81,82,83,84,85,86]. There are many opportunities for academics to partner with government, industry, and the third sector to translate research to practice to improve the lives of people rendered food insecure in rich nations.

The problem of food insecurity in developed countries is a growing problem with far reaching public health, social, and economic impacts. There will always be a need for food assistance to address emergency situations. But, this should not distract from the need to address the issue at its cause, in the words of Nelson Mandela, former President of South Africa, “*Overcoming poverty is not a gesture of charity. It is the protection of a fundamental human right, the right to dignity and a decent life*.” We call for governments to initiate actions to address the social determinants of food insecurity and to lead a coordinated, collaborative, and cooperative response to finding pathways out of food insecurity guided by the expertise, enthusiasm, and commitment of the third sector and the voices of those who have had the experience.
