# Correction: Pollard, C.M.; Booth, S. Food Insecurity and Hunger in Rich Countries—It Is Time for Action against Inequality. *Int. J. Environ. Res. Public Health* 2019, *16*, 1804

**DOI:** 10.3390/ijerph17072343

**Published:** 2020-03-30

**Authors:** Christina M. Pollard, Sue Booth

**Affiliations:** 1 Faculty of Health Science, School of Public Health, Curtin University, Perth 6845, Australia; 2 College of Medicine & Public Health, Flinders University, Adelaide 5000, Australia; sue.booth@flinders.edu.au

The authors wish to make the following correction to their paper published in the *International Journal of Environmental Research and Public Health* [1]: 

A mistake in the published text “In 2017, the absolute number of people affected by undernourishment or chronic food deprivation was estimated to be 821 million and 9 billion adults were overweight or obese.” 

Will be corrected to: “In 2017, the absolute number of people affected by undernourishment or chronic food deprivation was estimated to be 821 million and 672 million adults were obese.”

These changes have no material impact on the conclusions of our paper. We apologize to our readers.
